# The Impact of a Late Diagnosis: A Case of Charcot-Marie-Tooth Type 1

**DOI:** 10.7759/cureus.33727

**Published:** 2023-01-12

**Authors:** Fernando Albuquerque, Deolinda Cunha, Ana C Rodrigues, Rita Nunes, Filipe G Fernandes, Teresa Pipa, Ana Marques, Carla Moreira

**Affiliations:** 1 Family and Community Medicine, Unidade de Saúde Familiar (USF) Lusitana, Viseu, PRT

**Keywords:** charcot-marie-tooth, demyelinating diseases, pés cavus, genetic mutation, gene expression, congenital, sensorimotor neuropathy, neurology

## Abstract

Charcot-Marie-Tooth (CMT) is a hereditary motor and sensory neuropathy. The disease consists of a spectrum of inherited disorders caused by pathogenic variants in genes, which lead to multiple different clinical phenotypes. It is one of the most common inherited neuromuscular disorders.

This disease most commonly presents with symptoms of distal weakness and muscular atrophy, which then lead to foot drop and pés cavus. In this article, we describe the case of a patient who developed muscle atrophy and distal weakness over the course of his 52 years of life, leading to gait impairment and foot deformities. Subsequent investigation led to the acknowledgment of chronic axonal sensorimotor polyneuropathy and genetic identification of the disease's genotype, CMT type 1.

## Introduction

Charcot-Marie-Tooth (CMT) disease is a spectrum of inherited disorders caused by pathogenic variants in genes that are expressed in peripheral nerve myelin and/or axons. The overall estimated prevalence of CMT is 40 per 100,000 individuals, which varies from 10 to 82 per 100,000 individuals in different studies [1,2]. There are over 80 genes associated with CMT, although CMT types 1 (CMT1) and 2 (CMT2) represent, by far, the vast majority of patients [3,4]. CMT is associated with different pathogenic variant types, including whole-gene duplication, deletions, and point pathogenic variants [4]. The association of different pathogenic variants within the same gene is very common [4]. Presentation with various clinical phenotypes is also common [4].

The most frequent initial presentation of CMT is distal weakness and atrophy manifesting with foot drop and pés cavus [4]. Sensory symptoms are often present but usually less prominent [4]. Genetic testing is a key to confirming the diagnosis associated with electromyography (EMG) [4]. In this article, we report the case of a 52-year-old man who developed symptoms throughout his life and discuss how a late diagnosis impacted his quality of life.

## Case presentation

A 52-year-old male presented with a progressive history of gait claudication since adolescence and complained of distal muscle weakness in both lower limbs and progressive deformity of both feet on November 2021. He is divorced and lives with his current partner and his 12-year-old daughter.

He was submitted to surgical intervention on his left foot in 1987, but no records of the procedure are available, and the patient could not provide additional information. Besides this surgery, the patient was obese and suffered from dyslipidemia. He was medicated with atorvastatin.

His mother, a 77-year-old woman, had similar symptoms since early adulthood. Upon diagnosis, she was referred to secondary care to proceed with her study. His daughter, sister, grandparents, uncles, and aunts are all healthy and present no symptoms.

**Figure 1:**
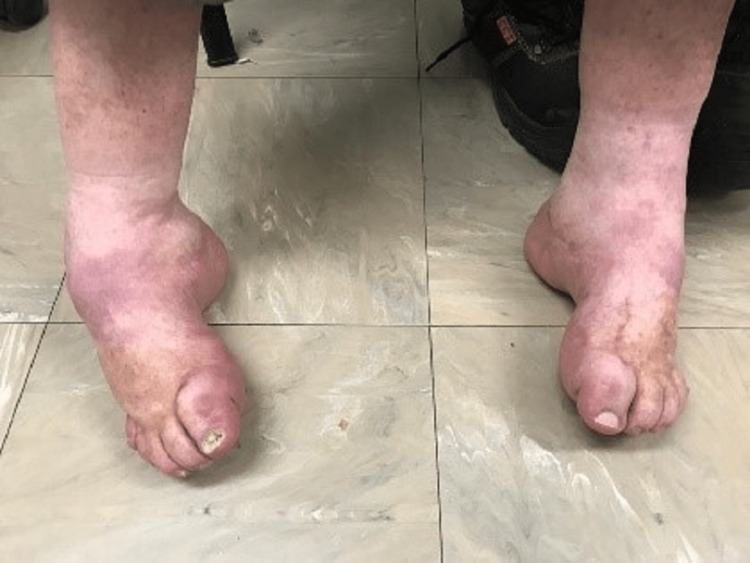
Pés cavus and equinovarus feet were identified at physical examination

At physical examination, pés cavus and equinovarus feet were identified. The patient presented without active movements on his toes and with severe calf atrophy (Figure 1). We also identified an areflexic flaccid tetraparesis, mostly in the distal muscle groups. These features combined made him bear all his weight on the lateral surface of both feet when walking, which he could only do with help of crutches.

We formulated the hypothesis of motor polyneuropathy and referred the patient to the specialties of neurology-physical medicine and rehabilitation for additional testing and prescription of orthotic devices to improve functionality, respectively.

He had an appointment with a physiatric in the same month, who prescribed orthopedic footwear. The patient received the footwear in April 2022 and immediately noticed an improvement in his daily activities.

In December 2021, he also had an appointment with a neurologist. An EMG was solicited, which revealed a very severe, chronic axonal sensorimotor polyneuropathy with demyelinating features and no conduction block (Tables 1-3).

**Table 1:** Sensitive electromyography (EMG) showing no sensitive potential of the right sural nerve and decreased velocities of the right medianus, right ulnaris, and right peroneus superficial sensory nerves Lat: Latency; Amp: Amplitude; dur: Duration; CV: Conduction velocity.

Sensitive				
	Lat	Amp	Dur	CV
	ms	mV	ms	m/s
Medianus Sensory Right				
Dig III - Wrist	6.16	1.39	5.1	21.3
Peroneus Superfic Sensory Right				
Leg - Ankle	4.87	0.68	3.7	18.9
Suralis Sensory Right				
Lat. Malleolus - Mid Lower Leg	--	--	--	
Ulnaris Sensory Right				
Dig V - Wrist	5.83	0.81	4.0	21.8

**Table 2:** Motor EMG showing no motor potential of the right peroneus and right tibialis motor nerves and decreased velocities of the right medianus and right ulnaris motor nerves EMG: Electromyography; Lat: Latency; Amp: Amplitude; CV: Conduction velocity; APB: Abductor pollicis brevis; EDB: Extensor digitorum brevis; ADM: Abductor digiti minimi; Bl: An error that occurred while transcripting the exam, which is not supoposed to be there; Abd hal: Abductor hallucis muscle.

Motor						
	Lat	Amp		Area		CV
	ms	mV	%	ms*mv	%	m/s
Motor Medianus Right						
Wrist - APB	11.3	1.18		4.5		
Elbow - Wrist	23.9	0.89	-24.6	2.5	-44.4	20.2
Motor Peroneus Right						
Ankle - EDB	--	--		--		
Bl. Knee - Ankle	--	--	--	--	--	--
Motor Tibialis Right						
Ankle - Abd hal	--	--		--		
Knee - Ankle	--	--	--	--	--	--
Motor Ulnaris Right						
Wrist - ADM	8.51	0.23		0.59		
Elbow - Wrist	19.3	0.16	-30.4	0.33	-44.1	20.3

**Table 3:** EMG findings showing fibrillations and scarce positive waves of the right tibialis anterior and right vastus medial muscles, with reduced recruitment EMG: Electromyography; Fib: Fibrillation potential; PSW: Positive sharp wave; IP: Interference pattern; MUP: Motor unit potential.

		Spontaneous activity		Voluntary activity					
Muscle	Interpretation	Fib	PSW	Amp	Dur	Poly	IP	Recruit	Notes
Right vastus med	Neurogenic	1+	0	+	+	+	-	Reduced	
Right tibialis anterior	Neurogenic	1+	1+	+	+	+	---	Reduced	1 MUP

Following these results, genetic testing was conducted, which demonstrated peripheral myelin protein 22 (PMP22) duplication (Table 4). As a result, the diagnosis of Charcot-Marie-Tooth type 1A (CMT1A) was confirmed, and the patient received counseling regarding the disease.

**Table 4:** Genetic test report showing peripheral myelin protein 22 (PMP22) duplication and confirming the diagnosis of Charcot-Marie-Tooth type 1A CMT1A: Charcot-Marie-Tooth type 1A.

Result
A heterozygotic duplication of the chromosomal region that comprehends the PMP22 gene was identified (17p12).
Interpretation
This result confirms the clinical diagnosis of Charcot-Marie-Tooth disease type 1A caused by duplication of 17p12.
The detected variant is a heterozygotic duplication that includes at least the PMP22 gene described in other patients with CMT1A.

## Discussion

Hereditary neuropathies are part of a heterogeneous group of diseases, with an insidious onset and progressive course of symptoms in common. CMT is the most common inherited peripheral neuropathy [5-7]. The major categories of CMT are CMT1 through 7 and an X-linked variant. Pathogenic variants in PMP22, myelin protein zero (MPZ), gap junction protein beta 1 (GJB1), and mitofusin 2 (MFN2) account for most cases, although numerous causative genes were identified [4]. CMT1 is characterized by peripheral nerve demyelination and an autosomal dominant inheritance. CMT1A is caused due to duplication of the PMP22 gene (the mutation with which the patient presented), which locates on chromosome 17 (17p11.2) and accounts for approximately 70%-80% of CMT1 cases [7-10].

This disease presents a varied spectrum of symptoms, ranging from asymptomatic patients to severely disabled ones, with symptoms beginning in the first or second decades of life [5,8]. Our patient presented symptoms since adolescence, as referred to in the literature. However, he maintained his daily activities and functionality, which delayed seeking health care. Therefore, it only came to our attention later in life when he could not do his daily activities due to the progression of the disease. The main features are a combination of lower motor neuron-type motor deficit as well as sensor signs and symptoms, which reflects sensory-motor neuropathy [5]. It typically starts with distal foot weakness, progressing in an ascending pattern [5]. Patients present a typical phenotype with pés cavus, hammer toes, high-arched feet, and distal muscle atrophies (peroneal and tibialis anterior), which may affect ambulation [5,8]. As the disease progresses, the proximal muscles of the lower limbs and intrinsic muscles of the hand become compromised as well [5,8]. Subjective changes in sensitivity are infrequent [8]. However, a neurologic exam may show a lower proprioceptive sense, followed by pain and temperature desensitization, with stocking and gloves distribution [5,8]. Osteotendinous reflexes are abolished or weaker, initially in the ankles and later in the patella and upper limbs [5,8].

CMT is diagnosed with EMG and sural nerve biopsy. EMG and nerve conduction studies are extremely helpful in the clinical classification of hereditary peripheral neuropathies. CMT1 presents with severe slowing of nerve conduction velocities (<38 m/s) and, in some patients, visible or enlarged nerves [6,7]. On the other hand, CMT2 presents with normal to near-normal nerve conduction velocities, no enlarged nerves, and reduced compound muscle action potential [6,7]. These results allow us to guide genetic testing. Sural nerve biopsy shows an onion-bulb formation and decreased myelinated fibers in CMT1 [6].

Patients with CMT disease have a slowly progressive neurodegenerative disease, so they benefit from periodic assessments. Orthotic devices and assistive equipment may improve the functionality of these patients, and physiotherapy and occupational therapy maintain the range of motion with benefits for their daily activities [2-5]. Some patients benefit from surgical intervention on the hands and feet [5].

Pharmacological treatment has an important impact on the quality of life. Neuropathy pain can be treated with antiepileptic drugs, such as gabapentin, pregabalin, and topiramate, or tricyclic antidepressants, such as amitriptyline [5]. Neurotoxic drugs, such as alcohol, should be avoided [5].

As CMT is a hereditary disease, genetic counseling is of utmost relevance as an affected parent with autosomal dominant has a risk of 50% of having a child with the same mutation [5]. Its penetration is still undetermined, which makes it impossible to know when an affected child will develop symptoms [5]. Without a molecular diagnosis, nerve conduction studies could help determine the diagnosis. Slowing of nerve conduction is detectable by age 2 to 5 [5]. Therefore, if a young adult has normal nerve conduction, the risk of developing the disease is negligible [5].

## Conclusions

In conclusion, the patient's quality of life improved as a result of our fast approach and referral of this medical problem as soon as it was identified. This case highlights the importance of family medicine in studying patients, not only as an individual but as part of a family. In this case, identifying this patient and his ancestry with similar symptomatology allowed us to stay more alert to his daughter. By doing this, we can refer her earlier than this patient. It also prompted an investigation of his mother, which is yet to be done.
